# Altered Gray Matter Volume and Functional Connectivity in Human Immunodeficiency Virus-Infected Adults

**DOI:** 10.3389/fnins.2020.601063

**Published:** 2020-12-03

**Authors:** Dan Liu, Cui Zhao, Wei Wang, Yuanyuan Wang, Ruili Li, Jun Sun, Jiaojiao Liu, Mingming Liu, Xu Zhang, Ying Liang, Hongjun Li

**Affiliations:** ^1^Department of Radiology, Beijing Youan Hospital, Capital Medical University, Beijing, China; ^2^School of Biomedical Engineering, Capital Medical University, Beijing, China; ^3^Department of Radiology, Beijing Second Hospital, Beijing, China; ^4^Physical Examination Center, Cangzhou Central Hospital, Hebei, China; ^5^Beijing University of Aeronautics and Astronautics, Beijing, China

**Keywords:** human immunodeficiency virus, HIV-associated neurocognitive disorder, structural MRI, resting-state functional MRI, functional connectivity

## Abstract

People living with human immunodeficiency virus (HIV) (PLWH) are at high risk of neurocognitive impairment. The pathogenesis of neurocognitive impairment remains unclear, and there is still no diagnostic biomarker. By coupling three-dimensional T1-weighted imaging and resting-state functional imaging, we explored structural and functional alterations in PLWH and examined whether such imaging alterations had the potential to denote neurocognitive function. A total of 98 PLWH and 47 seronegative controls aged 20–53 years were recruited. Structural alterations were first explored between HIV-negative controls and PLWH. Subsequently, brain regions showing gray matter alterations were used as seeds for separate whole-brain functional connectivity (FC) analysis. Finally, the relationships between imaging alterations and cognitive function were explored. PLWH suffered from thalamus, occipital lobe, and hippocampus/parahippocampus atrophy. Visual cortices in PLWH showed decreased anticorrelation with the posterior cingulate cortex and left angular gyrus of the default mode network. FC within the visual cortices (between the left calcarine and right calcarine) and in the thalamic prefrontal circuit and between the thalamus and somatosensory association cortex were also altered. In addition, FC between the left thalamus and right dorsolateral prefrontal cortex in the cognitively impaired group was significantly different from that in the cognitively normal group in PLWH. Partial correlation analysis uncorrected for multiple comparisons suggested that some imaging alterations can be associated with neurocognition. Our study supports the presence of brain atrophy and functional reconfiguration in PLWH. Imaging alterations can be associated with neurocognitive function. We hold that neuroimaging is a promising approach in evaluating PLWH and might have the potential to clarify the pathogenesis of HIV-associated neurocognitive disorder.

## Introduction

Human immunodeficiency virus (HIV) enters the central nervous system (CNS) soon after infection (Davis et al., 1992) and causes brain damage, including inflammation, microglial/macrophage activation, oligodendrocyte dysfunction, neural degeneration, and even apoptosis of neural cells (Zayyad and Spudich, 2015; Jensen et al., 2019; Rojas-Celis et al., 2019). Approximately 15–50% of people living with HIV (PLWH) show HIV-associated neurocognitive disorder (HAND) (Schouten et al., 2011). The prevalence varies according to the population studied and increases with age (Makinson et al., 2019). Methods used to define impairment also have an impact on the prevalence (Underwood et al., 2019). To date, the pathogenesis underlying HAND has not been fully characterized. It is believed that an indirect effect of chronic immune activation, inflammatory response (González-Scarano and Martín-García, 2005; Saylor et al., 2016) and a direct neurotoxic effect of viral protein (Nath, 2002), as well as vascular damage work together and may result in HAND in the combined antiretroviral therapy (cART) era (Zayyad and Spudich, 2015).

Neuroimaging is a promising approach to assess brain morphometric and functional changes that shows broad application prospects in the diagnosis and management of PLWH and may have the potential to shed light on the pathogenesis of HAND (Clifford and Ances, 2013; Saylor et al., 2016; Campbell et al., 2019). Previous structural MRI studies indicated that PLWH display widespread brain atrophy in the subcortical nucleus as well as the frontal, parietal, occipital, temporal, and cerebellar cortices and cortical thinning in the frontal and temporal lobes and cingulate cortex (Wilson et al., 2015; Underwood et al., 2017; Sanford et al., 2018a,b; Israel et al., 2019; Hassanzadeh-Behbahani et al., 2020). Diffuse abnormalities in white matter (WM) microstructure were also noticed, including reduced fractional anisotropy with increased mean and radial diffusivity (O’Connor et al., 2017; Underwood et al., 2017). Functional MRI (fMRI) indicated hyperactivation in task-related brain regions (Hakkers et al., 2017), disruption of functional connectivity (FC) within cortical networks, and corticostriatal networks in PLWH compared with controls (Thomas et al., 2013; Plessis et al., 2014; Ortega et al., 2015; Chaganti et al., 2017) and a reduction in resting cerebral blood flow (rCBF) in lenticular nuclei and the visual cortex (Ances et al., 2009). Positron emission tomography as molecular imaging provides valuable insight into the pathophysiology underlying HAND, and several studies reported perturbation of metabolism, neuroinflammation, abnormal function of neurotransmitters, and amyloid/tau protein deposition in HAND (Vera et al., 2016; Vera et al., 2017; Sinharay and Hammoud, 2019). In general, dopamine-modulated frontal-striatal circuit alteration was most consistently reported in previous studies and may play a part in the pathogenesis of HAND. Some researchers investigated the effects of cART using neuroimaging, and most of them reported improved characteristics in viral suppressed PLWH, including increased cortical thickness, no further structural deterioration, and improved FC (Ortega et al., 2015; Zhuang et al., 2017; Sanford et al., 2018a,b). The relationship between cognitive performance and neuroimaging is controversial. Whereas neuroimaging alterations in morphometry, diffusion, FC, and network topology were reported to be correlated with cognitive function (Wilson et al., 2015; Underwood et al., 2017; Haddow et al., 2019; Israel et al., 2019; Kato et al., 2020), there are also studies that failed to find any such associations (Thomas et al., 2013; Su et al., 2016; Wang et al., 2018).

In this study, by coupling voxel-based morphometry (VBM) analysis based on high-resolution T1-weighted MRI and seed-based whole-brain FC analysis based on resting-state fMRI (rs-fMRI), we investigated brain morphometric and FC alterations in PLWH and further explored the association between imaging alterations and neurocognitive function.

## Materials and Methods

### Participants

A total of 98 PLWH and 47 seronegative controls (HIV-negative controls) aged 20–53 years were recruited in our study. The inclusion criteria for both groups were as follows: right-handed and able to give informed consent. For the PLWH, only those infected by homosexual sex were included. The exclusion criteria for both groups were as follows: (1) medical or neuropsychiatric illnesses that might confound the study; (2) previous head injury with loss of consciousness longer than 30 min; (3) substance abuse; and (4) MR contraindication. Clinical data were obtained from medical records and are presented in Table 1. Among the 98 PLWH, 63 were tested for current viral load, 90 were tested for hepatitis B virus and hepatitis C virus, and 98 were tested for syphilis. This study was approved by the institutional review boards, and all the participants provided written informed consent in accordance with the Declaration of Helsinki.

**TABLE 1:** Demographics, clinical information, and neurocognitive performance.

**Variable**	**Control (*n* = 46)**	**GDS normal (*n* = 55)**	**GDS impaired (*n* = 36)**	***P*-value**
Age (mean ± *SD*)	34.4 ± 6.7	32.0 ± 7.2	30.8 ± 7.1	0.057^a^
Male, *n* (%)	42 (91.3%)	54 (98.2%)	36 (100.0%)	0.089^d^
Education years (mean ± *SD*)	/	13.0 ± 3.7	14.3 ± 2.7	0.060^b^
CD4 + T (mean ± *SD*)	/	473.9 ± 233.3	496.2 ± 208.3	0.643^b^
CD4/CD8 (mean ± *SD*)	/	0.6 ± 0.4	0.6 ± 0.5	0.982^b^
TND, *n* (%)	/	30 (85.7%)	18 (81.8%)	0.722^d^
BMI (mean ± *SD*)	/	21.1 ± 2.7	22.4 ± 2.6	0.031^b^
Hepatitis B virus infection, *n* (%)	/	13 (26.5%)	9 (26.5%)	1.000^c^
Hepatitis C virus infection, *n* (%)	/	1 (2.0%)	0 (0.0%)	1.000^d^
Syphilis, *n* (%)	/	19 (34.5%)	16 (44.4%)	0.383^c^
TIV (mean ± *SD*)	1,507.7 ± 132.8	1,528.2 ± 120.1	1,553.0 ± 100.9	0.240^a^
Neurocognitive performance *T* scores (mean ± *SD*)				
Speed of information processing	/	48.4 ± 7.2	40.8 ± 9.5	< 0.001^b^
Memory (learning and recall)	/	46.8 ± 6.5	39.1 ± 7.7	< 0.001^b^
Abstraction/executive	/	58.4 ± 9.1	51.9 ± 8.6	0.001^b^
Attention/working memory	/	44.8 ± 7.2	38.2 ± 6.9	< 0.001^b^
Fine motor skills	/	48.4 ± 7.7	41.2 ± 11.8	0.001^b^
verbal and language	/	48.7 ± 7.0	39.8 ± 9.6	< 0.001^b^
GDS	/	0.1 ± 0.1	0.8 ± 0.8	< 0.001^b^

*TND, virus not detectable; BMI, body mass index; TIV, total intracranial volume; GDS, global deficit score.P-value for comparison among three groups (age, male%, TIV) and between GDS normal and GDS impaired subgroups.Statistical tests including: ^*a*^one-way ANOVA; ^*b*^t-test; ^*c*^chi-square test; ^*d*^Fisher’s exact test.*

### Neurocognitive Tests

Six cognitive domains were assessed in PLWH using a set of neurocognitive tests normed for age, sex, education level, and scale of residence area (Shi et al., 2015), including (1) speed of information processing [trail-making test part A (TMT A)]; (2) memory, including learning and recall [Hopkins verbal learning test-revised (HVLT-R) and brief visuospatial memory test-revised (BVMT-R)]; (3) abstraction and executive function [Wisconsin Card Sorting Test 64-card version (WCST-64)]; (4) attention and working memory [continuous performance test-identical pairs (CPT-IP), Wechsler memory scale-III (WMS-III), and Paced Auditory Serial Addition Test (PASAT)]; (5) fine motor skills (Grooved Pegboard Test); and (6) verbal and language (animal naming test). The global deficit score (GDS) approach was used to determine cognitive status in PLWH, as it considers both the number and severity of cognitive deficits in performance throughout the overall test battery (Blackstone et al., 2012). A cutoff of 0.5 was applied to classify PLWH into GDS normal and GDS impaired subgroups according to previous studies (Carey et al., 2004).

Only PLWH were provided with neurocognitive tests; among them, four men and three women were not tested.

### Neuroimaging Acquisition

All images were acquired using a Siemens Trio Tim 3T scanner with a 32-channel head coil. Foam pads were used to restrict head movement. All participants were asked to lie relaxed and not to think of any particular things with their eyes closed without falling asleep.

Structural images were collected using a sagittal magnetization prepared gradient-echo (MPRAGE) sequence [repetition time (TR)/echo time (TE)/inversion time (TI) = 1,900/2.52/900 ms; acquisition matrix = 256 ^∗^ 246; field of view = 250 ^∗^ 250; flip angle = 9 degrees; voxel size = 1 mm ^∗^ 1 mm ^∗^ 1 mm].

Whole-brain rs-fMRI data were collected utilizing a gradient echo single-shot echo planar imaging (EPI) sequence (TR/TE = 2,000/30 ms; resolution matrix = 64 ^∗^ 64; voxel size = 3.5 mm ^∗^ 3.5 mm ^∗^ 3.5 mm; flip angle = 90°) with 35 axial slices and 240 time points.

One HIV-negative control was excluded for inadequate imaging acquisition.

### Image Preprocessing

All images were first reoriented using Statistical Parametric Mapping 12 (SPM12)^1^ to align images with standard space as closely as possible and set the origin to be close to the anterior commissure. T1-weighted images were preprocessed using computational anatomy toolbox 12 (CAT12)^2^ within SPM12 using MATLAB R2013b for voxel-based morphometry (VBM) analysis. As parts of the images showed inhomogeneity, in the segmentation module, we selected the strength of the SPM inhomogeneity correction as strong, SPM processing accuracy as high, and strength of the local adaptive segmentation as medium. Images were normalized to the Montreal Neurological Institute (MNI) 152 space using the diffeomorphic anatomical registration through exponentiated lie algebra (DARTEL) algorithm (Ashburner, 2007). Data quality was subsequently checked by running the check data quality module in CAT12. We visually inspected each normalized bias-corrected volume to remove volumes with artifacts and unsatisfactory orientations, and modulated normalized gray matter (GM) segments were checked to identify outliers as well as sample homogeneity. No participant was excluded. Total intracranial volume (TIV) was estimated. Finally, all the modulated normalized GM segments were smoothed with an 8 mm full-width at half-maximum (FWHM) kernel.

Resting-state fMRI was preprocessed with Data Processing and Analysis for Brain Imaging (DPABI V4.2) (Yan et al., 2016). The first 10 timepoints were removed to guarantee signal stability. Slice timing correction was performed with the timing of the middle slice as a reference. The time series of each participant was realigned using a six-parameter (rigid body) linear transformation with a two-pass procedure (first realigned to the initial image and then to the mean of the image). After realignment, T1-weighted images were coregistered to mean functional images using 6 degrees of freedom linear transformation and subsequently segmented into GM, WM, and cerebrospinal fluid (CSF). DARTEL was used to compute transformations from individual native space to MNI space. The Friston 24-parameter head motion model was used to regress out head motion effects (Friston et al., 1996; Yan et al., 2013). Mean framewise displacement (FD), which was derived from Jenkinson’s relative root mean square algorithm, was used to reduce the residual effects of motion in group analyses (Jenkinson et al., 2002). Linear trends were regressed to reduce drift in the signal, and CSF and WM signals were regressed as covariates through linear regression to reduce respiratory and cardiac effects. Global signal regression (GSR) was also applied because it can reduce unwanted global confounds, such as vascular effects, respiration, and movement; enhance the detection of system-specific correlations; and improve the correspondence between resting-state correlations and anatomy (Fox et al., 2009; Zhu et al., 2015; Murphy and Fox, 2017). The temporal bandpass filter (0.01∼0.1 Hz) was used to remove the low-frequency drifts and high-frequency physiological noise.

Nine HIV-negative controls and seven PLWH were excluded for excessive head motion (mean FD > 0.2 mm), and two PLWH were excluded for poor image registration. A group binary mask was created that allowed 90% of participants to have the voxel in each participant’s brain coverage for statistical analysis.

Cortical renderings were visualized with BrainNet Viewer (Xia et al., 2013).

### Functional Connectivity Analysis

The bilateral thalamus, right calcarine, right cuneus, bilateral lingual, left hippocampus, left parahippocampus, and left fusiform, which exhibited GM alterations, were defined as regions of interest (ROI). These ROI masks were extracted from the Automated Anatomical Labeling (AAL) atlas. The average time series of voxels in each ROI was extracted. Pearson’s correlation analysis was used to calculate the temporal correlation between the ROI and each voxel of the brain. Fisher’s r- to -z transformation was used to improve data normality. An isotropic Gaussian kernel at 4 mm FWHM was used for spatial smoothing.

### Statistical Analyses

Statistical tests conducted on demographics, clinical information, and neurocognitive function comparisons included one-way analysis of variance (ANOVA) (age, TIV), two-sample *t*-test [education, CD4 + T cell count, CD4/CD8, body mass index (BMI), neurocognitive function in each of the six domains], chi-square test (hepatitis B virus, syphilis), and Fisher’s exact test (sex, percentage of viral load not detectable, hepatitis C virus). Group differences (PLWH and HIV-negative controls) in brain volume and FC were identified using a two-sample *t*-test. Correction for multiple comparisons used Gaussian random field (GRF) correction, with statistical significance defined by voxel level *P* < 0.001 and cluster level *P* < 0.05 (two-tailed). Brain volume and FC differences among HIV-negative controls, GDS normal groups, and GDS impaired groups were identified using a univariate general linear model, and *post hoc* multiple comparisons were conducted using the least significant difference (LSD) test. Partial correlation was applied to explore the relationship between each cognitive domain, GDS, and imaging alterations, with a false discovery rate (FDR) of *P* < 0.05 after correction for multiple comparisons.

Statistical analyses were performed using IBM Statistics version 22. A *P* < 0.05 was considered statistically significant.

## Results

### Participants

Demographics and clinical characteristics are summarized in Table 1. There were no significant differences between the PLWH subgroups in education, current CD4 + T cell count, CD4/CD8, percentage of undetectable viral load, or percentage of hepatitis B virus/hepatitis C virus/syphilis coinfection. The GDS impaired group showed a higher BMI than the GDS normal group (*T* = −2.2, *P* = 0.031). Fisher’s exact test showed no significant difference in sex. One-way ANOVA indicated no age and TIV differences among the HIV-negative controls, GDS normal, and GDS impaired groups (*F* = 2.9, *P* = 0.057; *F* = 1.4, *P* = 0.240).

In our study, 39.6% of PLWH were neurocognitively impaired according to the GDS approach. Compared with GDS normal participants, GDS impaired participants showed significantly decreased T scores in all six cognitive domains and GDS, as shown in Table 1.

### Difference in Gray Matter Volume Between HIV-Negative Controls and People Living With HIV

**FIGURE 1:**
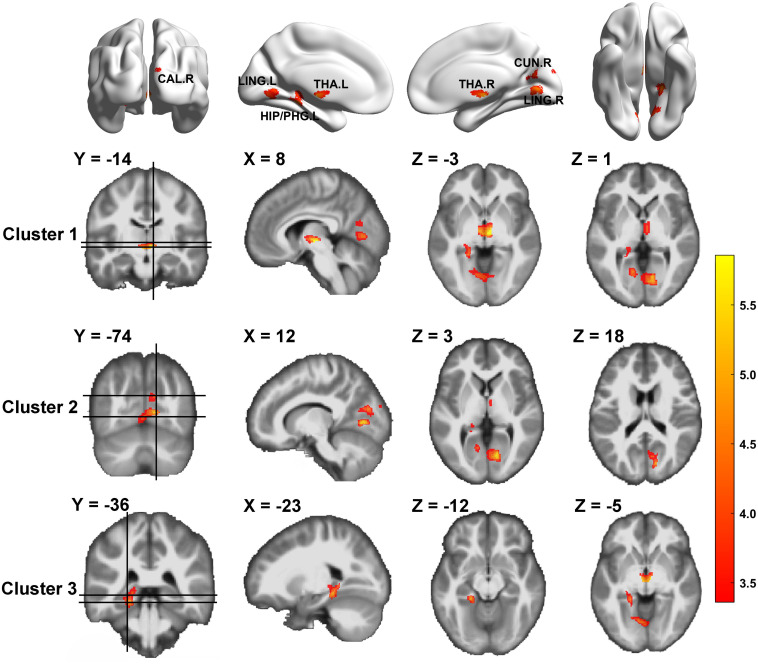
Gray matter volume differences between HIV-negative controls and people living with HIV (PLWH) based on voxel-based morphometry (VBM) analysis. Brain regions with significantly reduced gray matter colored by T-statistic (red/yellow). Corrected for multiple comparisons [Gaussian random field (GRF) correction, voxel level *P* < 0.001, cluster level *P* < 0.05, two-tailed].

We compared GM volume (GMV) between HIV-negative controls and PLWH (Table 2 and Figure 1). The GMV of the bilateral thalamus, right calcarine (extended to the right lingual), right cuneus, left lingual, left hippocampus, and left parahippocampus (extended to the left lingual, left fusiform) in PLWH were significantly reduced compared with HIV-negative controls after controlling for age, sex, and TIV (GRF corrected, voxel level *P* < 0.001, cluster level *P* < 0.05, two-tailed).

**TABLE 2:** Gray matter volume differences between HIV-negative controls and PLWH.

**Structure**		**L/R**	**Volumes (mm^3^)**	**Coordinates**			***T*-value**	***P*-value**
				**X**	**Y**	**Z**		
Cluster 1	Thalamus	R	756.0	8	−14	−3	5.9	<0.001
	Thalamus	L		−5	−14	−5	5.2	<0.001
Cluster 2	Calcarine	R	7,519.5	12	−74	3	5.5	<0.001
	Cuneus	R		17	−74	38	4.9	<0.001
	Lingual	L		−9	−68	−6	4.5	<0.001
Cluster 3	Parahippocampus	L	2,575.1	−21	−38	−11	5.4	<0.001
	Hippocampus	L		−18	−36	0	4.3	<0.001

*Coordinates (X, Y, Z) refer to the peak MNI coordinates of brain regions with peak intensity. Corrected for multiple comparisons (GRF correction, voxel level P < 0.001, cluster level P < 0.05, two-tailed).GRF, Gaussian random field; MNI, Montreal Neurological Institute; PLWH, people living with HIV.*

### Differences in Seed-Based Whole-Brain Functional Connectivity

**FIGURE 2:**
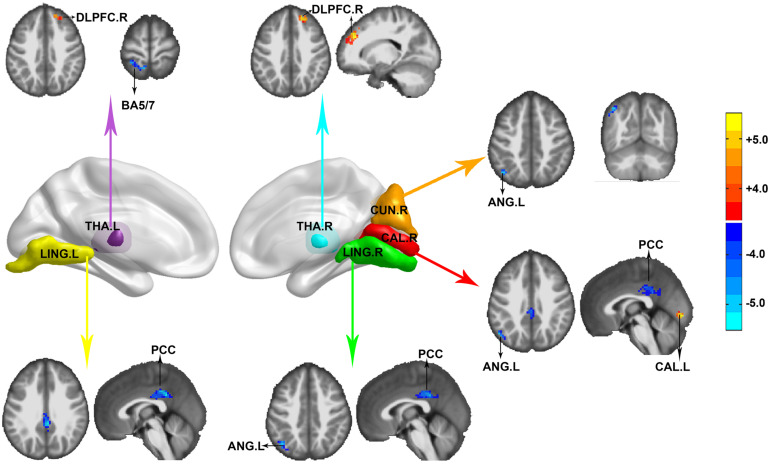
Functional connectivity (FC) differences between HIV-negative controls and people living with HIV (PLWH). Seeds were extracted according to the Automated Anatomical Labeling (AAL) atlas. THA.L, left thalamus (purple); LING.L, left lingual (yellow); CUN.R, right cuneus (orange); CAL.R, right calcarine (red); LING.R, right lingual (green); and THA.R, right thalamus (cyan). Corrected for multiple comparisons [Gaussian random field (GRF) correction, voxel level *P* < 0.001, cluster level *P* < 0.05, two-tailed]. Color bar indicates corrected *T*-values.

FC was calculated and compared between HIV-negative controls and PLWH after controlling for age, sex, and head motion. Reduction of anticorrelation was noticed among the left lingual, right calcarine, right lingual, and posterior cingulate cortex (PCC). FC between the right calcarine, right cuneus, and left angular (BA39) was also reduced. The PCC and left angular detected in our study were located in the default mode network (DMN). The positive correlation within the visual network (between the right calcarine and left calcarine) was also reduced. The left thalamus showed decreased FC in the ipsilateral precuneus/superior parietal lobe (BA5, BA7) and right dorsolateral prefrontal cortex (DLPFC), whereas the right thalamus showed decreased FC in the right DLPFC in PLWH compared with HIV-negative controls. No significant HIV effect was observed in the FC of the left hippocampus/parahippocampus and left fusiform (GRF correction, voxel level *P* < 0.001, cluster level *P* < 0.05, two-tailed), as shown in Figure 2.

### Relationship Between Cognitive Function and Imaging Alterations

**FIGURE 3:**
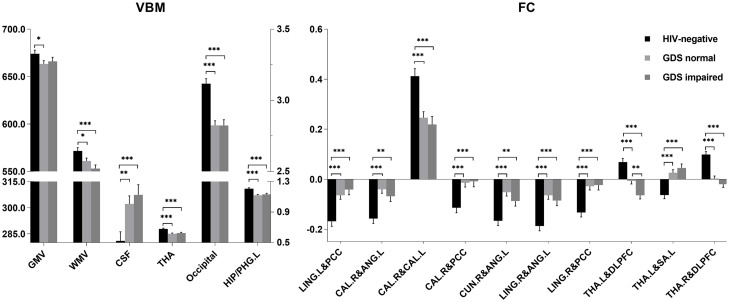
Volume (ml) and functional connectivity (FC) (Z-score) differences among HIV-negative controls, global deficit score (GDS) normal, and GDS impaired groups. For voxel-based morphometry (VBM) analysis, the left Y-axis is the volume of gray matter (GM), white matter (WM), and cerebrospinal fluid (CSF), and the right Y-axis is the volume of thalamus (THA), occipital, and left hippocampus/parahippocampus (HIP/PHG.L). ^∗^*P* < 0.05; ^∗∗^*P* < 0.01; ^∗∗∗^*P* < 0.001. Least significant difference (LSD) for multiple comparison correction. LING.L, left lingual; PCC, posterior cingulate cortex; CAL.R, right calcarine; CAL.L, left calcarine; CUN.R, right cuneus; ANG.L, left angular; LING.R, right lingual; DLPFC, dorsal lateral prefrontal cortex; THA.L, left thalamus; THA.R, right thalamus; SA.L, left somatosensory association cortex.

**FIGURE 4:**
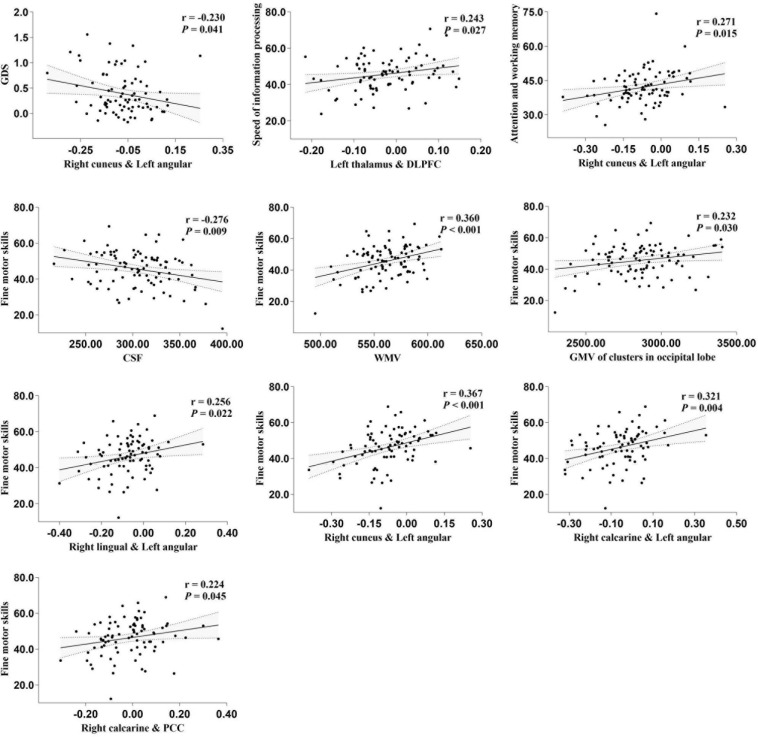
partial correlation between specific cognitive domains, global deficit score (GDS), and imaging alterations. The areas between two dotted curves indicate the 95% confidence interval. All variables shown above were unstandardized residuals plus their own mean value.

Total GMV, WM volume (WMV), CSF volume, and clusters showed that differences in GMV and FC due to serostatus were further compared to explore whether cognition status had an effect on these imaging parameters. No significant differences were detected between PLWH subgroups after controlling for age, sex, TIV (only for GMV comparisons), or head motion (only for FC comparisons), except that the anticorrelation between the left thalamus and right DLPFC was significantly increased in the GDS impaired group compared with the GDS normal group (Figure 3). Partial correlation analysis conducted between specific cognitive domains, GDS, and imaging alterations indicated that right cuneus-left angular FC was negatively correlated with GDS (*r* = −0.230, *P* = 0.041). Speed of information processing was positively correlated with left thalamus-DLPFC FC (*r* = 0.243, *P* = 0.030). Attention and working memory were positively correlated with right cuneus-left angular FC (*r* = 0.271, *P* = 0.015). Fine motor skills were positively related with total WMV (*r* = 0.360, *P* < 0.001), GMV of the cluster in the occipital lobe (*r* = 0.232, *P* = 0.03), right lingual-left angular FC (*r* = 0.256, *P* = 0.022), right cuneus-left angular FC (*r* = 0.367, *P* < 0.001), right calcarine-angular FC (*r* = 0.321, *P* = 0.004), and right calcarine-PCC FC (*r* = 0.224, *P* = 0.045), whereas total CSF volume showed a negative correlation (*r* = −0.276, *P* = 0.009) (Figure 4). However, after correction for multiple comparisons with a FDR of *P* < 0.05, these findings were not significant.

## Discussion

A combination of VBM analysis and resting-state FC in our study indicated that PLWH suffered from thalamus, occipital lobe, and hippocampus/parahippocampus atrophy. The visual cortices in PLWH showed decreased anticorrelation with the PCC and left angular of the DMN. The FC within the visual network was also reduced. Alterations in FC in the thalamic prefrontal circuit between the thalamus and somatosensory association cortex were also detected. In addition, FC between the left thalamus and right DLPFC in the GDS impaired group was significantly different from that in the GDS normal group. Partial correlation analysis uncorrected for multiple comparisons suggested that certain imaging alterations can be associated with neurocognition. However, after correction for multiple comparisons, these results were not significant. We discussed partial analysis without FDR correlation in the following sections to discern all the possible relationships between imaging alterations and cognitive function.

Glutamate is the most abundant neurotransmitter in the nervous system. Aberrant metabolism of this neurotransmitter plays an important role in the development of HAND (Ernst et al., 2010; Nedelcovych et al., 2019). Previous studies suggested that macrophages and activated astrocytes can produce tumor necrosis factor (TNF) and other inflammatory cytokines, resulting in increased glutamate release and reduced glutamate uptake by astrocytes. The subsequently high levels of glutamate resulted in uncontrolled Ca^2+^ influx, which led to neuron degeneration and apoptosis (Zayyad and Spudich, 2015). Neuron loss is rare in the cART era but not in degeneration, and synaptodendritic degenerative changes have been reported to be associated with HAND (Guha et al., 2018). We presume that the injury to glutamate neurons might account for the scattered brain atrophy and decreased neural synchronous activity detected in our study.

The thalamus is a central relay station of sensory signals and the integration center of the CNS. Several previous studies delineated thalamus atrophy in PLWH (Castillo et al., 2018; Sanford et al., 2018a,b). In our study, thalamus atrophy was mainly located in the medial nucleus (e.g., mediodorsal nucleus, MD), which contributes principal subcortical input to the DLPFC (Popken et al., 2000). Moreover, a growing body of evidence suggests a causal relationship between MD-PFC abnormalities and cognitive deficits, including working memory, behavioral flexibility, goal-directed behavior, and recollection (Danet et al., 2017; Parnaudeau et al., 2018). In our study, FC between the left thalamus and right DLPFC in the GDS impaired group was significantly different from that in the GDS normal group and positively correlated with the speed of information processing. We speculate that neuroimaging alterations can be associated with neurocognition.

The ventral posterolateral nucleus and MD of the thalamus also participate in pain processing (Fenton et al., 2015). The same is true for somatosensory association cortex (Witting et al., 2001). It is worth noting that HIV-associated sensory neuropathy, a common neuropathy, is characterized by neuropathic pain, sensory loss, and minimal weakness (Aziz-Donnelly and Harrison, 2017). Both the periphery and CNS are involved in this neuropathy. A study evaluated brain morphometry abnormalities within the pain matrix and detected smaller surface areas in the prefrontal pars triangularis, anterior cingulate cortex, smaller thalamus, and putamen in PLWH (Castillo et al., 2018). Further research is needed to test whether the abnormalities delineated in our study contribute to the pathogenesis of this condition.

GM atrophy and FC reduction within the visual network were reported in PLWH (Wang et al., 2011; Underwood et al., 2017; Egbert et al., 2018). In addition, poorer fine motor skills were reported to correlate with reduced occipital cortex volume, lower CBF in the occipital cortex, and smaller supratentorial WMV (Haddow et al., 2019; Kato et al., 2020). Neuroinflammation, rCBF reduction, and lower electroencephalogram (EEG) rhythms were also reported in the occipital lobe in PLWH (Babiloni et al., 2014; Wiesman et al., 2018; Haddow et al., 2019). Evidence from neurocognitive assessment suggested episodic memory, visual attention, visuoperception, and visual working memory deficits in PLWH (Woods et al., 2009), which might indicate abnormalities in the occipital lobe. A cytopathology study examined brain tissue in the temporal, parietal, and occipital lobes and found that the calcarine cortex (primary visual area) had the greatest neuronal loss of 30% (Everall et al., 1993).

Reduced FC between the cortices of the visual network and PCC and the left angular of the DMN is relevant to other studies reporting FC reduction within the DMN and between the DMN and other networks in PLWH (Thomas et al., 2013; Ortega et al., 2015; Ann et al., 2016; Samboju et al., 2018). Moreover, the detected correlation with fine motor skills, attention and working memory, and GDS might suggest that compromised internetwork integration and communication impair cognitive function. Reduced FC between the visual network and DMN was also previously reported in elderly people (with a mean age of 60.2 ± 12.8 years) (Onoda et al., 2012). As our participants were relatively young (32.5 ± 7.0 years) and there was no significant difference across groups, the FC reduction observed in our study is unlikely due to aging. Whether the internetwork FC reduction suggests premature brain aging still requires further study.

The lower left hippocampus/parahippocampus volume detected in our cohort is consistent with other studies (Wilson et al., 2015). An autopsy study focused on the basal ganglia and hippocampus showed that neuroinflammation continued in cART-treated patients, particularly in the hippocampus (Anthony et al., 2005). In another neuropathological study, Sá et al. (2000) found that the total number of neurons in the hippocampus was not different from that in controls, but cell atrophy was detected in patients with AIDS, which supports the findings of our study from a pathologic perspective. Our study also suggested the decoupling of FC and GM atrophy in the hippocampus/parahippocampus. A similar phenomenon was reported in studies coupling WMV and diffusion in PLWH (Underwood et al., 2017) as well as studies focused on other disease models (Bernhardt et al., 2016; Peer et al., 2017).

There are several limitations in our current study. First, the lack of more detailed clinical and neurocognitive measures in seronegative controls limited its comparison to PLWH. In addition, we only orally asked for any medical history of mental disorders as an exclusion criterion, and it would be more rigorous to collect psychiatric symptoms based on neuropsychiatric testing. Second, some antiretroviral drugs have been reported to have neurotoxicity and cause neurodegenerative changes, neuropsychiatric symptoms, and brain volume reductions (O’Halloran et al., 2019). In our study, a combination of two nucleoside reverse transcriptase inhibitors (NRTIs) (usually tenofovir disoproxil fumarate and lamivudine) and one non-nucleoside reverse transcriptase inhibitor (NNRTI) (usually efavirenz) made up a free cART regimen. Additional caution should be taken as efavirenz has been proven to be neurotoxic (Ciccarelli et al., 2011). Moreover, the exact regimes and whether regimes were switched in each PLWH were not clear. Thus, we cannot eliminate such confounding factors caused by drugs. Third, the nature of the cross-sectional design makes it subject to inherent bias, and a longitudinal study by our group is now in progress for a more powerful, objective analysis.

Our study indicates that PLWH suffered from brain atrophy and functional reconfiguration, and imaging alterations can be associated with neurocognition. We hold that neuroimaging is a promising approach in evaluating PLWH and helps to elucidate the pathogenesis of HAND. Future longitudinal studies with large samples, more detailed clinical information, and neurocognitive information aimed at PLWH (both cART naive and on cART) are needed to clarify existing confounds and further increase our understanding of the pathogenesis of HAND.

## Data Availability Statement

The original contributions presented in the study are included in the article/supplementary material, further inquiries can be directed to the corresponding author/s.

## Ethics Statement

The studies involving human participants were reviewed and approved by the Institutional Review Boards of Beijing YouAn Hospital. The patients/participants provided their written informed consent to participate in this study.

## Author Contributions

HL and DL contributed to conception and design of the study. RL, WW, JL, YW, and ML recruited participants and organized the database. JS and CZ performed parts of the statistical analysis. DL collected parts of participants’ information, analyzed the statistics, and wrote the first draft of the manuscript. All authors contributed to manuscript revision, read, and approved the submitted version.

## Conflict of Interest

The authors declare that the research was conducted in the absence of any commercial or financial relationships that could be construed as a potential conflict of interest.

## Footnotes

1 http://www.fil.ion.ucl.ac.uk/spm/software/spm12/

2 http://www.neuro.uni-jena.de/cat/
